# Blast Exposure Leads to Accelerated Cellular Senescence in the Rat Brain

**DOI:** 10.3389/fneur.2020.00438

**Published:** 2020-05-21

**Authors:** Peethambaran Arun, Franco Rossetti, Donna M. Wilder, Sujith Sajja, Stephen A. Van Albert, Ying Wang, Irene D. Gist, Joseph B. Long

**Affiliations:** Blast-Induced Neurotrauma Branch, Center for Military Psychiatry and Neuroscience, Walter Reed Army Institute of Research, Silver Spring, MD, United States

**Keywords:** blast exposure, traumatic brain injury, aging, senescence, SA-β-gal, SMP-30, p21

## Abstract

Blast-induced traumatic brain injury (bTBI) is one of the major causes of persistent disabilities in Service Members, and a history of bTBI has been identified as a primary risk factor for developing age-associated neurodegenerative diseases. Clinical observations of several military blast casualties have revealed a rapid age-related loss of white matter integrity in the brain. In the present study, we have tested the effect of single and tightly coupled repeated blasts on cellular senescence in the rat brain. Isoflurane-anesthetized rats were exposed to either a single or 2 closely coupled blasts in an advanced blast simulator. Rats were euthanized and brains were collected at 24 h, 1 month and 1 year post-blast to determine senescence-associated-β-galactosidase (SA-β-gal) activity in the cells using senescence marker stain. Single and repeated blast exposures resulted in significantly increased senescence marker staining in several neuroanatomical structures, including cortex, auditory cortex, dorsal lateral thalamic nucleus, geniculate nucleus, superior colliculus, ventral thalamic nucleus and hippocampus. In general, the increases in SA-β-gal activity were more pronounced at 1 month than at 24 h or 1 year post-blast and were also greater after repeated than single blast exposures. Real-time quantitative RT-PCR analysis revealed decreased levels of mRNA for senescence marker protein-30 (SMP-30) and increased mRNA levels for p21 (cyclin dependent kinase inhibitor 1A, CDKN1A), two other related protein markers of cellular senescence. The increased senescence observed in some of these affected brain structures may be implicated in several long-term sequelae after exposure to blast, including memory disruptions and impairments in movement, auditory and ocular functions.

## Introduction

The incidence of blast-induced traumatic brain injury (bTBI) in the military increased significantly after the introduction of improvised explosive devices, with recent reports that 80% of mild TBI cases are related to blast exposure (1). The symptoms of mild TBI include headache, dizziness, fatigue, fogginess, as well as impairments in cognitive, vestibular, oculomotor, and psychological functions (2–5). Blast exposure is also known to cause acute and chronic neurobehavioral abnormalities whose severities increase with greater blast intensity (overpressure) and number of blast exposures. The acute effects of mild TBI resulting from explosive blast typically resolve within 1 to 3 weeks, but chronic symptoms of TBI develop in 15 to 30% of the cases (3, 6).

Both clinical and pre-clinical observations have prompted suggestions that bTBI yields a predisposition to age-related neurodegenerative disorders (7–12). Neuropathological evaluations in blast victims and in mice exposed to blast revealed that blast exposure promotes chronic traumatic encephalopathy (12). Acute and chronic development of tauopathy has been reported after blast exposure (8, 13), in which the phosphorylation of *Tau* protein disrupts microtubule assembly in neurons yielding tauopathy characterized by the formation of neurofibrillary tangles seen in neurodegenerative disorders such as Alzheimer's disease (AD) (14–16). Brain injury has been proposed as a factor enhancing the likelihood of early onset or acceleration of AD (9, 17). Although the neuropathology of AD is associated with aging, no studies have yet definitively illustrated whether brain injury after blast exposure accelerate the aging process.

Blast exposure has been reported to cause chronic white matter abnormalities which are associated with long-term memory impairments (18, 19). Initial clinical observations using diffusion tensor imaging to evaluate military victims of blast revealed a rapid age-related loss of white matter integrity in the brain, and the severity of the changes increased with number of exposures (19). In a concurrent study, loss of myelin integrity was similarly observed in primary blast casualties with and without mild TBI symptoms (18). Even though it is known that white matter integrity declines with aging, no further studies were carried out to determine whether the aging of brain cells after blast was associated with loss of white matter integrity.

Senescence-associated β-galactosidase (SA-β-gal) is a lysosomal enzyme expressed only in cells undergoing senescence processes and it is not normally expressed in presenescent, quiescent or immortal cells. It hydrolyzes β-galactosides to monosaccharides and the enzyme is active even at acidic pH (6.0). SA-β-gal activity has been widely used as a reliable marker of cellular senescence in brain (20–22) and other organs (23, 24). In the present study, using an advanced blast simulator (ABS) to expose animals to single and tightly coupled repeated blasts, we have carried out postmortem evaluation of SA-β-gal activity in different anatomical regions of the brain at various time points up to 1 year post-blast exposures to determine whether blast exposure accelerate cellular senescence, an indicator of aging processes.

## Materials and Methods

### Animals

All animal experiments were conducted in accordance with the Animal Welfare Act and other federal statutes and regulations relating to animals and experiments involving animals, and adhered to principles stated in the Guide for the Care and Use of Laboratory Animals (NRC Publication 2011 edition) using an Institutional Animal Care and Use Committee approved protocol. Male Sprague Dawley rats, 9–10 weeks old that weighed 300–350 g (Charles River Laboratories, Wilmington, MA) were housed at 20–22°C (12 h light/dark cycle). Rats were given free access to nutritious rat chow (Prolab IsoPro RMH 3000 from LabDiet, St. Louis, MO) and water *ad libitum* till 1 month after the blast exposure, when they reached a body weight of 400–450 g. We restricted diet for all rats including sham controls after 1 month so that the weight of the rats maintained between 450 and 500 g until the completion of the study (1 year). Body weights were recorded 3 days a week and adjustments were made in the quantity of diet to maintain body weights within this range. This diet restriction was required since, although not reported here, these animals underwent neurobehavioral functional tests reported by us elsewhere (25) and the weight gain otherwise resulting from feeding *ad libitum* adversely impacts performance on the neurobehavioral tests.

### Primary Blast Exposure

The ABS described previously was used for the study (25, 26). For blast exposure, the rats were anesthetized with 4% isoflurane for 8 min and secured in a longitudinal (i.e., rat facing the oncoming shockwave) prone orientation in the test section of the ABS. To produce moderate TBI in rats in these experiments, we used Valmax membranes yielding peak positive static pressures of ~19 psi with a positive phase duration of 4–5 ms. For tightly coupled repeated blast exposures, the rats were exposed to two 19 psi blast overpressure waves separated by 2 min as described earlier (25). The sham control rats were handles and exposed to 4% isoflurane anesthesia for 8 min, but were not subjected to blast exposure. After blast exposure, the rats were euthanized at 24 h, 1 month, or 1 year.

### Senescence Marker Staining

In order to determine whether blast exposure increases brain aging, we used senescence detection kits (BioVision, Milpitas, CA) according to the manufacturer's instructions. The kit stains only cells expressing senescence-associated β-galactosidase (SA-β-gal) enzyme and won't stain presenescent, quiescent or immortal cells. Briefly, at each time point after blast exposure, the animals were anesthetized by inhalationally administering 5 % isoflurane for 6 min and then were transcardially perfused first with normal saline followed by 4% paraformaldehyde. The brains were collected and post-fixed in 4% paraformaldehyde for 6 h followed by cryopreservation using 20% sucrose immersion overnight and finally stored in 30% sucrose. For tissue staining using the kit, 30 μm coronal brain sections were prepared using a cryostat. The sections were incubated overnight at 37°C with the senescence detection reagent. The sections were mounted and mosaic (12 × 14) pictures of different brain regions were taken using an Olympus BX61 microscope (Olympus Corporation, Center Valley, PA) and Stereo Investigator virtual image tool (MBF Biosciences, Williston, VT). The blue color developed inside the cells, which is a measure of SA-β-gal enzyme activity, was used for quantitation using densitometry. The densitometry analysis, to measure the density of blue coloration developed due to SA-β-gal enzyme activity, was performed using the Image-Pro Premier software (Media Cybernetics Inc., Rockville, MD). For densitometry measurements, those cells stained intensely were included and those cells that showed <50% of maximum staining intensity were excluded. A total of 6 shams, 5 single blast exposed, and 4 repeated blast exposed rats were used at each time point for senescence staining.

### Quantitative Real-Time Polymerase Chain Reaction (qRT-PCR)

The differential expressions of senescence marker protein-30 (SMP-30) and p21 (cyclin dependent kinase inhibitor 1A, CDKN1A) were determined in the brain cortex at 1 month post-blast using qRT-PCR. We selected the 1 month time point since the activity of SA-β-gal was the highest compared to 24 h and 1 year. We chose brain cortex for these measurements since among brain regions the activity of SA-β-gal was greatest in the cortex at both 24 h and 1 month after single and repeated blast exposures. Total RNA was extracted from the brain cortex using the RNeasy mini kit (Qiagen, Germantown, MD). Equal amounts of RNA were reverse transcribed into cDNA using RT^2^ first strand kit (Qiagen, Germantown, MD). qRT-PCR was performed using the RT^2^ SYBR green reagents in the QuantStudio 6 Flex qPCR system (Life Technologies, Grand Island, NY) using the proprietary primers from Qiagen (SMP-30, Cat. No: PPR44609A; p21, Cat. No: PPR06378B; β-actin, Cat. No: PPR06570C), and β-actin was used as an internal control. The relative gene expression was analyzed using the threshold cycle 2^−ΔΔCt^ method. A total of 6 rats per group were used for analysis and the results are presented as fold changes compared to control groups.

### Statistical Analysis

Statistical analysis was carried out by Two-way Analysis of Variance followed by Tukey's *post-hoc* test using HSD multiple comparisons (GraphPad Prism 6 software). The density values of the blue stain were expressed as mean ± standard error of the mean (SEM). For each time point, the density values of all three treatment groups were compared to each other. A *p* < 0.05 was considered significant.

## Results

### Blast Exposure Increased the Activity of SA-β-gal in Different Regions of the Brain

#### Cortex

**Figure 1:**
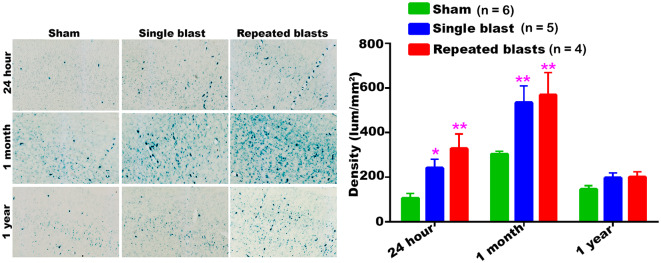
Activity of SA-β-gal in the motor cortex at different intervals post-blast exposures. Density values are expressed as mean ± SEM. Values of all three groups were compared to each other at each time point for statistical significant differences. *Blast exposed groups at each time point were compared to corresponding sham controls (*p < 0.05; **p < 0.01; *n* = 4–6).

In the cerebral cortex, significantly increased activity of SA-β-gal was observed at 24 h and 1 month after single and repeated blast exposures compared to sham controls (Figure 1). No statistically significant differences were observed between single and repeated blast exposed rats at any of the three time points evaluated. Compared to 24 h assessment, all the rats including sham controls showed increased activity of SA-β-gal at 1 month, but showed activity levels returning to those seen at 24 h by 1 year.

#### Auditory Cortex

**Figure 2:**
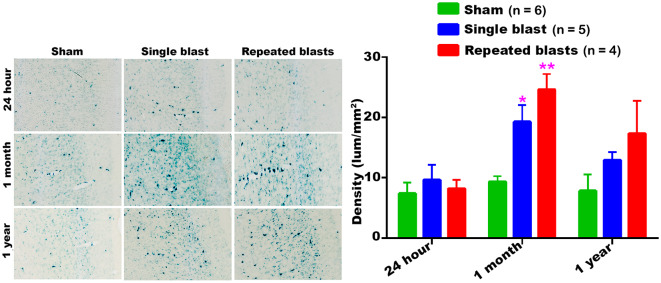
SA-β-gal activity in the auditory cortex at different intervals post-blast exposures. Density values are expressed as mean ± SEM. Values of all three groups were compared to each other at each time point for statistical significance. *Blast exposed groups at each time point were compared to corresponding sham controls (*p < 0.05; **p < 0.01; *n* = 4–6).

Compared to sham controls, rats exposed to single and repeated blasts showed increased activity of SA-β-gal in the auditory cortex at 1 month post-blast (Figure 2). In all the rats, including sham controls, the maximum activity of SA-β-gal was observed at 1 month. No significant differences were observed between the single and repeated blast exposed groups.

#### Dorsolateral Thalamus

**Figure 3:**
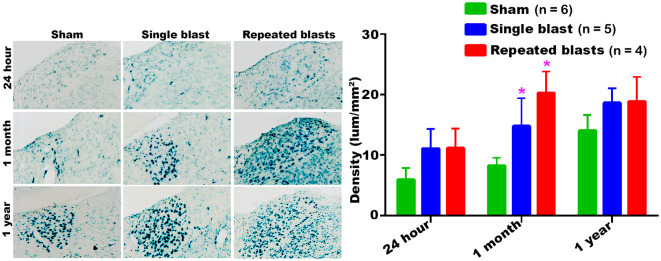
Dorsolateral thalamus showing the differential activity of SA-β-gal at different intervals post-blast exposures. Density values are expressed as mean ± SEM. Values of all three groups were compared to each other at each time point for statistical significance. *Blast exposed groups at each time point were compared to corresponding sham controls (*p < 0.05; *n* = 4–6).

Compared to sham controls, rats exposed to single and repeated blasts showed a statistically significant increase in the activity of SA-β-gal in the dorsolateral thalamus at 1 month and a trend toward increased activity at other times evaluated (Figure 3). At no time were statistically significant differences observed between the single and repeated blast treatment groups, and sham control rats showed a sustained increase in SA-β-gal activity across the observation times.

#### Superior Colliculus

**Figure 4:**
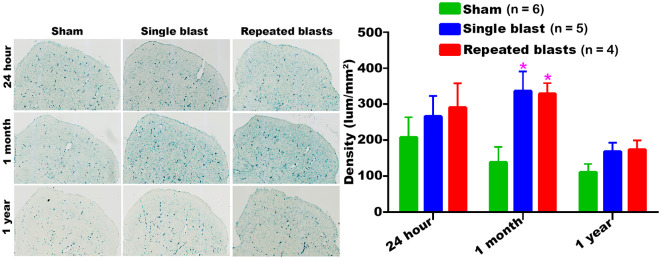
Activity of SA-β-gal in the superior colliculus at different intervals post-blast exposures. Density values are expressed as mean ± SEM. Values of all three groups were compared to each other at each time point for statistical significance. *Blast exposed groups at each time point were compared to corresponding sham controls (*p < 0.05; *n* = 4–6).

SA-β-gal activity in the superior colliculus of sham controls showed a sustained decrease from 24 h to 1 year (Figure 4). Compared to sham controls, rats exposed to single and repeated blasts showed a statistically significant increase in the activity of SA-β-gal in the superior colliculus at 1 month and a trend toward increased activity at other time points evaluated (Figure 4). No statistically significant differences were observed between the single and repeated blast treatment groups at any time.

#### Geniculate Nucleus

**Figure 5:**
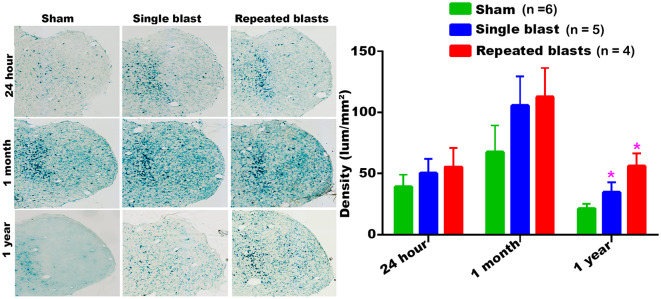
Geniculate nucleus showing the differential activity of SA-β-gal at different intervals post-blast exposures. Density values are expressed as mean ± SEM. Values of all three groups were compared to each other at each time point for statistical significance. *Blast exposed groups at each time point were compared to corresponding sham controls (*p < 0.05; *n* = 4–6).

Compared to sham controls, rats exposed to single and repeated blasts showed a statistically significant increase in the activity of SA-β-gal in the superior colliculus at 1 year and a trend toward increased activity at other time points evaluated (Figure 5). Once again, no statistically significant differences were observed between single and repeated blast exposed animals. Compared to 24 h measurements, sham control animals showed an upward trend in the activity of SA-β-gal at 1 month which by 1 year returned back below the 24 h levels.

#### Ventral Thalamic Nucleus

**Figure 6:**
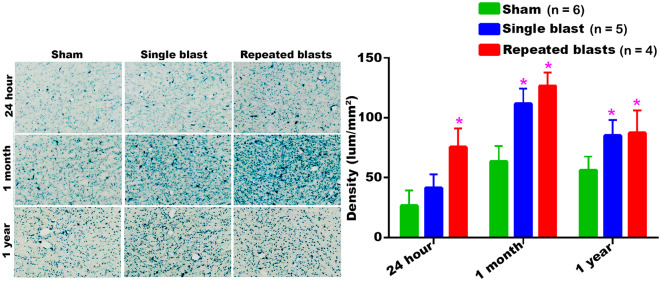
SA-β-gal activity in the ventral thalamic nucleus at different intervals post-blast exposures. Density values are expressed as mean ± SEM. Values of all three groups were compared to each other at each time point for statistical significance. *Blast exposed groups at each time point were compared to corresponding sham controls (*p < 0.05; *n* = 4–6).

Compared to sham treatment, activity of SA-β-gal in the ventral thalamic nucleus was significantly increased at all the three time points evaluated after repeated blast exposures (Figure 6). In the case of single blast exposed group, statistically significant increase in SA-β-gal was observed at 1 month and 1 year compared to sham controls (Figure 6). Once again, no statistically significant differences were observed between single and repeated blast exposed groups at all three evaluation times and the maximum activity of SA-β-gal was observed in the ventral thalamic nucleus of sham animals at 1 month.

#### Hippocampus

**Figure 7:**
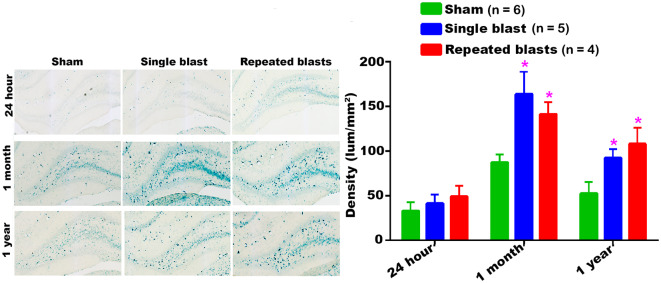
Activity of SA-β-gal in the hippocampus at different intervals post-blast exposures. Density values are expressed as mean ± SEM. Values of all three groups were compared to each other at each time point for statistical significance. *Blast exposed groups at each time point were compared to corresponding sham controls (*p < 0.05; *n* = 4–6).

Single and repeated blast exposures significantly increased the activity of SA-β-gal in the hippocampus at 1 month and 1 year post-blast (Figure 7) and no statistically significant differences were observed between single and repeated blast exposure groups. As was seen in other brain regions, the hippocampus again showed maximum activity of SA-β-gal at 1 month.

### Blast Exposure Leads to Differential Expression of Both SMP-30 and p21 mRNAs in the Cerebral Cortex

**Figure 8:**
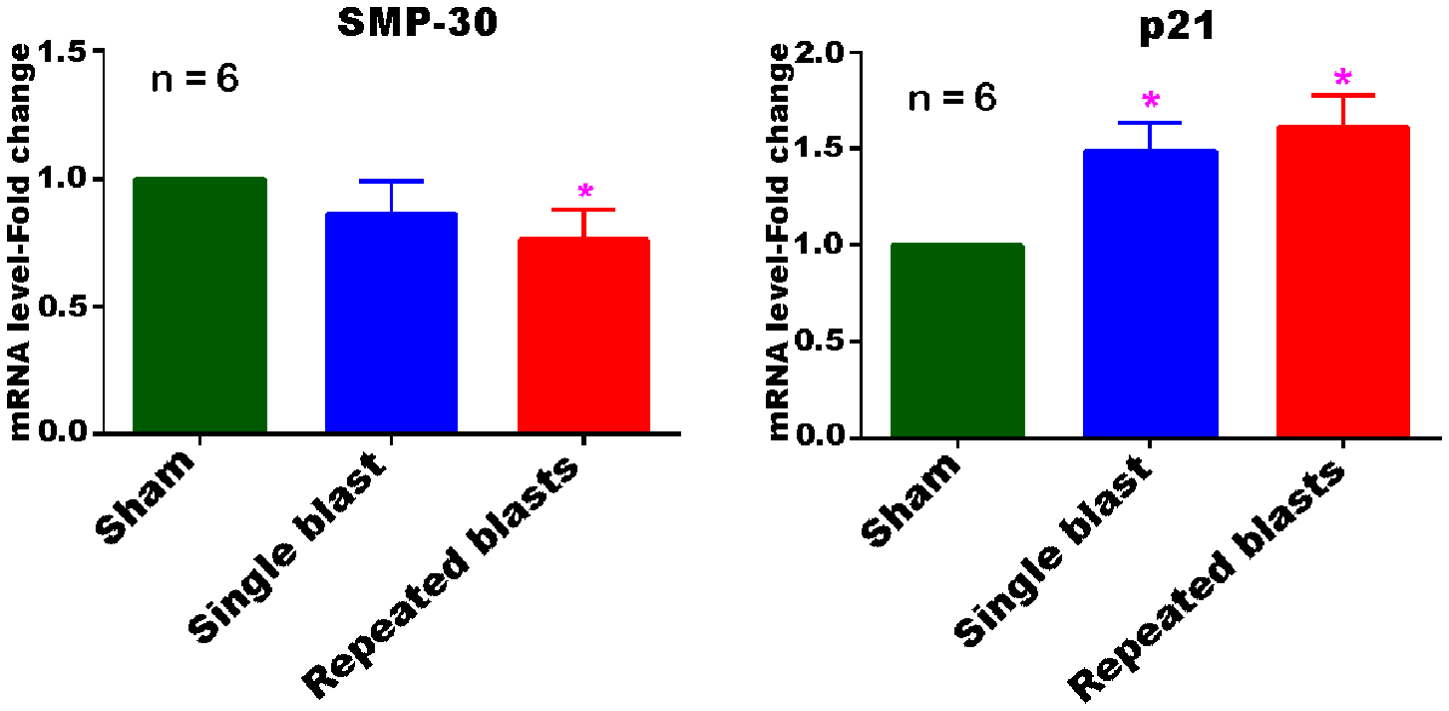
Differential expression of SMP-30 and p21 mRNAs in the cerebral cortex at 1 month post-blast exposures. Values of the blast exposed groups are expressed as mean ± SEM. Fold changes in the expressions of mRNAs of blast exposed groups were compared to those of sham controls (*p < 0.05; *n* = 6).

Evaluation of the cerebral cortex tissue using qRT-PCR analysis revealed differential expression of both SMP-30 and p21 mRNAs at 1 month after repeated blast exposures (Figure 8). The mRNA levels of SMP-30 were decreased and p21 mRNA increased significantly in the cortex at 1 month after repeated blast exposures. In rats exposed to a single blast, the mRNA levels of p21 increased significantly, whereas those of SMP-30 did not significantly decrease, although the levels were generally lower than in shams. Neither mRNA was expressed differently between single and repeated blast exposed groups.

## Discussion

The results of this preclinical study support previous indirect clinical observations and speculations that blast exposure may accelerate brain aging processes (18, 19). Those clinical findings focused on the integrity of white matter, which is known to diminish with age, whereas our preclinical evaluation was mostly on gray matter regions. Using diffusion tensor imaging (DTI), a large number of military service members were evaluated to determine whether blast exposure affected the integrity of brain white matter (19). The diffusion contrast measures, fractional anisotropy and radial diffusivity, showed that white matter integrity in blast-exposed veterans and pre-deployed service members was significantly lower than was recorded in military personnel without a history of blast exposure, and further suggested that the degree of the loss of white matter integrity was directly proportional to the severity and number of blast exposures (19). Loss of myelin integrity after primary blast exposure in victims with and without mild TBI symptoms was similarly described in a concurrent study employing DTI and comparing the same diffusion contrast measures, fractional anisotropy and radial diffusivity (18). In that report, all blast exposed victims had decreased fractional anisotropy and increased radial diffusivity irrespective of having mild TBI symptoms, suggesting that primary blast exposure affects the integrity of myelin and may thereby accelerate brain aging processes. Their results also suggest that the absence of clear TBI symptoms following primary blast may not accurately reflect or predict the severity of underlying brain injury. Even though it is known that loss of white matter integrity occurs with aging, no further studies have connected cellular aging processes with loss of white matter integrity after primary blast exposure. In the present study, using an advanced blast simulator (ABS) to expose animals to single and tightly coupled repeated blasts, we have carried out postmortem evaluations of various brain regions at different time points up to 1 year post-blast exposures and have shown that blast exposure accelerates cellular senescence, an indicator of aging processes, in different parts of the brain.

Our data showed that blast exposure, especially repeated blast exposure, significantly increased the activity of SA-β-gal in different regions of the rat brain in a time dependent manner (Figures 1–7). We did not observe uniformly increased activity of SA-β-gal throughout the brain; rather, changes were restricted to several specific regions of the brain and occurred at different times post-injury. Here, we have shown only those brain regions that showed a significant increase in the activity of SA-β-gal after blast exposure. In most of the brain regions evaluated, sham control rats showed an increase in the activity of SA-β-gal at 1 month compared to that measured at 24 h, possibly reflecting a normal increase in cellular senescence with age. However, compared to the 1 month measurements, the activity of SA-β-gal in sham controls decreased at 1 year and was comparable to that recorded at 24 h in multiple brain regions with the exception of the dorsolateral thalamus, which showed a continued increase, and the superior colliculus, which showed a sustained decrease from 24 h to 1 year. As described in the methods, diet restriction was initiated at 1 month and was continued through 1 year to prevent weight gain during that time and allow neurobehavioral assessments to be performed (25). Since diet/calorie restriction has been shown to inhibit cellular senescence in pre-clinical and clinical studies (27, 28), it is quite possible that the decrease in cellular senescence in some of the brain regions at 1 year compared to 1 month or 24 h was due to the diet restriction. The decreased activity of SA-β-gal at 1 year could also possibly be due to the removal of senescent cells, by the immune system. Cells undergoing senescence are reported to display a pro-inflammatory phenotype and the immune system typically clears such cells (29, 30). It is also possible that the initiation and progression of cellular senescence in the dorsolateral thalamus and superior colliculus could simply differ in timing and scope from that occurring in the other brain regions evaluated.

Significant increases in the activity of SA-β-gal were observed at 24 h and 1 month post-blast in the cerebral cortex and ventral thalamic nucleus. These findings are potentially important in view of the observation that victims of blast exposure often suffer from significant movement and balance dysfunctions (31, 32). In uninjured individuals, movement and balance functions deteriorate with aging and if blast exposure hastens the senescence of cells in these brain regions, it may account at least in part for hastened and worsened balance problems observed among blast casualties (32). Cerebral cortex is one of the frequently evaluated brain regions after blast exposure, but not much information is available on the effect of blast exposure on the ventral thalamic nucleus. Single and repeated blast exposures have been shown to cause blood-brain barrier disruption, oxidative stress, pro-inflammatory processes, and phosphorylation of tau proteins in the cerebral cortex (12, 33–35). Inflammation and oxidative stress are known to be associated with cellular senescence (36, 37).

We have observed increased activity of SA-β-gal in the dorsolateral thalamic nucleus, geniculate and superior colliculus suggesting that senescence process is accelerated in those brain regions after blast exposure. All three of these brain regions play critical roles in the processing of visual signals in the brain; consequently, it is possible that accelerated cellular senescence in these neuroanatomical structures might promote early deterioration of vision. Preclinical studies have shown that blast exposure leads to axonal fiber degeneration in the superior colliculus and geniculate resulting in ocular dysfunctions (38, 39). Exposure to blast waves has been implicated as the major cause of visual dysfunction in veterans involved in combat operations, with deficits being primarily attributed to ocular injury (40, 41). Perturbation of brain visual signal processing centers by shock waves also contribute to these deficits (2, 42–44). A long-term study carried out in patients with blast-induced mild TBI without immediate eye injuries revealed that 68% nevertheless had visual dysfunctions, confirming the prominent role of injuries to these structures (43).

Large numbers of cells in the auditory cortex showed increased SA-β-gal activity at 1 month after single and repeated blast exposures, although immediate changes observed at day 1 post-blast were modest. Since the auditory cortex is the most important brain region involved in auditory signal processing, the rapid cellular senescence in this brain region may have implications concerning the chronic auditory dysfunctions that are widely seen after blast exposures. Preclinical studies have shown acute and chronic neuronal degeneration in the auditory cortex after blast exposure (45). Electrophysiological evaluations of the auditory cortex after blast exposure showed spontaneous firing of neurons up to 3 months post-blast indicative of blast-induced tinnitus (46). Blast-induced auditory dysfunctions are considered as the most prevalent disabilities resulting from Operation Iraqi Freedom and Operation Enduring Freedom (47), with up to 62% of blast injured patients exhibiting hearing loss and tinnitus (48). Hoffer et al. evaluated US Marines with mild traumatic brain injuries (mTBI) from combat-related blasts and found that prevalence of hearing loss was 33% in acute patients and 49% in chronic patients (32). In many victims of blast exposure, severe auditory dysfunctions occur despite an intact tympanic membrane, suggesting that hearing loss can result from both inner ear injury and central auditory processing defects (CAPD) (47). It is notable that SA-β-gal activity in auditory cortex was increased by exposure to blast, but it is presently unclear what if any role this might play in chronic CAPD.

The hippocampus is widely studied after blast exposure since it is the vital neuroanatomical structure in the brain involved in short and long-term memory signal processing. Oxidative stress, neuroinflammation, phosphorylation of tau protein and axonal degeneration were reported in the hippocampus after blast exposure (41, 49–52). The significantly increased activity of SA-β-gal in the hippocampus at 1 month and 1 year after single and repeated blast exposures in the present study is noteworthy in view of the fact that blast exposed victims experience short and long-term memory problems (53–57) and acute and chronic memory deficits have been reported in a number of different animal models of blast TBI (58, 59). In particular, it has been shown that casualties as a result of exposures to blast within a 10 meter radius will likely develop memory problems in the later stages of life (60).

Although the increase in the activity of SA-β-gal is a reliable marker of cellular senescence, it was necessary to examine a few other known indicators of cellular senescence to rule out the possibility that blast exposure is only increasing the activity of SA-β-gal and not leading to the senescence process. The qRT-PCR results obtained in the cerebral cortex at 1 month after single and repeated blast exposures are consistent with the increased activity of SA-β-gal observed in the cortex at 1 month, suggesting that the increased activity of SA-β-gal after blast exposure is associated with cellular senescence. The expression of p21 is widely used as a marker of senescence in combination with the activity of SA-β-gal (61–63). The increased expression of p21 in the cells undergoing senescence leads to cell-cycle arrest through inhibition of cyclin-dependent kinases. Upregulation of the p21 pathway of cellular senescence in human neuroblastoma cells has been shown to trigger cellular senescence and accumulation of α-synuclein, the protein that accumulates in the brain of patients with Parkinson's disease (64). Compared to p21, only very few studies have applied SMP-30 as a marker of senescence along with SA-β-gal (65, 66). SMP-30 protein expression is known to decrease in the cells with age (67, 68). The combination of decreased mRNA levels of SMP-30 and increased mRNA levels of p21 along with increased activity of SA-β-gal protein in the cerebral cortex at 1 month after repeated blast exposures strongly suggest that the affected cells are undergoing accelerated senescence process after blast exposures. It is presently unclear from these measurements whether neurons or glial cells or both are undergoing senescence; further studies are required to distinguish specific cell type(s) undergoing senescence in this and other regions of the brain after blast exposure.

Increased senescence after brain injury has been reported previously in other experimental animal models (21, 69). In a very recent study, brain injury resulting from controlled cortical impact (CCI) in mice caused increased activity of SA-β-gal in the ipsilateral cerebral hemisphere on days 4, 7, and 14 with a maximum increase on the 7th day, along with significant changes on the contralateral side as well (21). Significantly increased expression of p21 was observed in both neurons and microglia in the ipsilateral side on days 1, 4, and 14, indicating that both neurons and microglia might be undergoing senescence (21). In addition, increased expression of p16, another known marker of senescence, was observed in astrocytes in the ipsilateral cerebrum on days 1, 4, and 14, prompting the suggestion that astrocytes also may be undergoing accelerated senescence after injury (21). Another recent study using CCI in mice showed that several markers of senescence, including p21 and p16, were increased in microglia at 72 h after the injury, providing additional indications that accelerated senescence after brain injury is not limited to neurons (69). Both of these studies included evaluations only at acute and subacute times after brain injury and were limited to observations mostly in the injured hemisphere. In the present study, we have greatly expanded this timeline and have observed indications of accelerated cellular senescence through 1 year following single and repeated blast exposures. Further studies are now warranted and required to determine the specific cell types undergoing the accelerated senescence process and to discern the mechanisms triggering cellular senescence after blast exposure.

## Data Availability Statement

All datasets generated for this study are included in the article/supplementary material.

## Ethics Statement

The animal study was reviewed and approved by WRAIR-IACUC.

## Author Contributions

PA and JL designed the experiments. DW and SV performed the blast experiments. FR performed senescence staining and analyses. YW and IG performed qRT-PCR. PA and SS performed data analysis. PA and JL wrote the manuscript.

## Conflict of Interest

The authors declare that the research was conducted in the absence of any commercial or financial relationships that could be construed as a potential conflict of interest.
